# Association between serum albumin levels and erectile dysfunction in American Adults: A cross-sectional study from NHANES 2001–2004

**DOI:** 10.1371/journal.pone.0318147

**Published:** 2025-02-06

**Authors:** Haibin Wen, Zhenyu Lan, Xueming Liang, Huabin Su, Yuqi Qin

**Affiliations:** 1 Department of Nephrology, Jiang bin Hospital of Guangxi Zhuang Autonomous Region, Nanning, China; 2 Department of Nutrition, Jiang bin Hospital of Guangxi Zhuang Autonomous Region, Nanning, China; 3 Department of Scientific Research, Jiang bin Hospital of Guangxi Zhuang Autonomous Region, Nanning, China; 4 Medical Management Service Guidance Center of Guangxi Zhuang Autonomous Region, Nanning, China; Tehran University of Medical Sciences, IRAN, ISLAMIC REPUBLIC OF

## Abstract

**Background:**

The aim of this study is to investigate the association between Serum Albumin Levels (ALB) and erectile dysfunction (ED) within the U.S. general population.

**Methods:**

We conducted a cross-sectional analysis using data from the National Health and Nutrition Examination Survey (NHANES) 2001–2004 cycles. Serum albumin was analyzed both as a continuous variable and categorized into quartiles. Erectile dysfunction (ED) was assessed via self-reported questionnaires. The association between serum albumin and erectile dysfunction was evaluated using weighted logistic regression models across four models: (1) Crude model (unadjusted); (2) Model 1: Adjusted for age, race, poverty-to-income ratio (PIR), marital status, education level, and body mass index (BMI); (3) Model 2: Adjusted for factors in Model 1 plus physical activity, smoking status, drinking status, and Healthy Eating Index (HEI-2015); (4) Model 3: Adjusted for factors in Model 2 plus remaining potential covariates. A generalized additive model (GAM) was employed to examine non-linear associations, followed by subgroup analyses and interaction tests.

**Results:**

A total of 2925 participants were included in the study, of which 747 were diagnosed with ED. After adjusting for all covariates, a significant negative association was found between ALB and ED (OR: 0.53, 95% CI: 0.29–0.97, P = 0.04). Higher ALB quartiles were significantly correlated with a decreased risk of ED [Q4 vs. Q1: OR: 0.56 (0.35–0.90), P =  0.02; P for trend =  0.03]. The GAM and smoothed curve fit indicated a linear relationship between ALB and the risk of ED. Stratified and interaction tests further substantiated the inverse relationship between ALB and ED prevalence.

**Conclusions:**

This study revealed an inverse association between ALB and ED. Therefore, it is important for clinicians to recognize the assessment of ALB in patients.

## 1. Introduction

Erectile dysfunction (ED) is characterized by the persistent or recurrent inability to achieve or maintain an erection sufficient for satisfactory sexual performance [1]. ED not only deteriorates the quality of male sexual life and affects fertility, but it also frequently signals potential cardiovascular diseases or other serious health conditions [2–4]. It is estimated that approximately 20% of men in the United States experience some form of ED, with the incidence increasing markedly with age [5,6].

The etiology of ED is multifactorial, involving complex vascular, neurogenic, endocrine, and psychosocial components [7,8]. Prior research has established links between ED and various medical and lifestyle factors, including obesity, diabetes, hypertension, and depression [9–11]. Recently, serum albumin levels have also been implicated in the risk of ED development [12]. As a principal transport protein, serum albumin not only reflects nutritional status but is also a crucial marker for evaluating inflammatory and endothelial functions [13].

Previous cross-sectional studies have shown an association between low serum albumin levels and increased ED risk [14,15]. However, these studies are often constrained by limited sample sizes and a lack of population diversity. An analysis using nationally representative epidemiological data, such as the National Health and Nutrition Examination Survey (NHANES), could enhance our understanding of this association and its broader clinical implications.

ED is both a common genitourinary disorder and a marker of severe conditions including cardiovascular and cerebrovascular diseases. Therefore, identifying modifiable risk factors for ED is essential for early prevention [16,17]. Population-based analysis of serum albumin and ED may help identify high-risk individuals and guide prevention strategies. This study aims to evaluate the association between serum albumin levels and ED using NHANES data from 2001 to 2004. We hypothesize that lower serum albumin levels are positively associated with the prevalence of ED.

## 2. Materials and methods

### 2.1. Study design and participants

The analyzed data were extracted from the NHANES database, specifically from the 2001–2002 and 2003–2004 survey cycles. We utilized data from NHANES 2001–2004 cycles as these were the only cycles that included erectile dysfunction assessment in the questionnaire. This approach aligns with previous ED studies using NHANES data [18,19]. The National Health and Nutrition Examination Survey (NHANES) is a series of cross-sectional surveys designed to assess the health and nutritional status of the non-institutionalized civilian population in the United States [20]. This survey integrates demographic, socioeconomic, dietary, and health-related data collected through face-to-face interviews, physical examinations, and extensive laboratory tests. The comprehensive methodology and value of NHANES have been previously described in detail [21,22].

**Fig 1:**
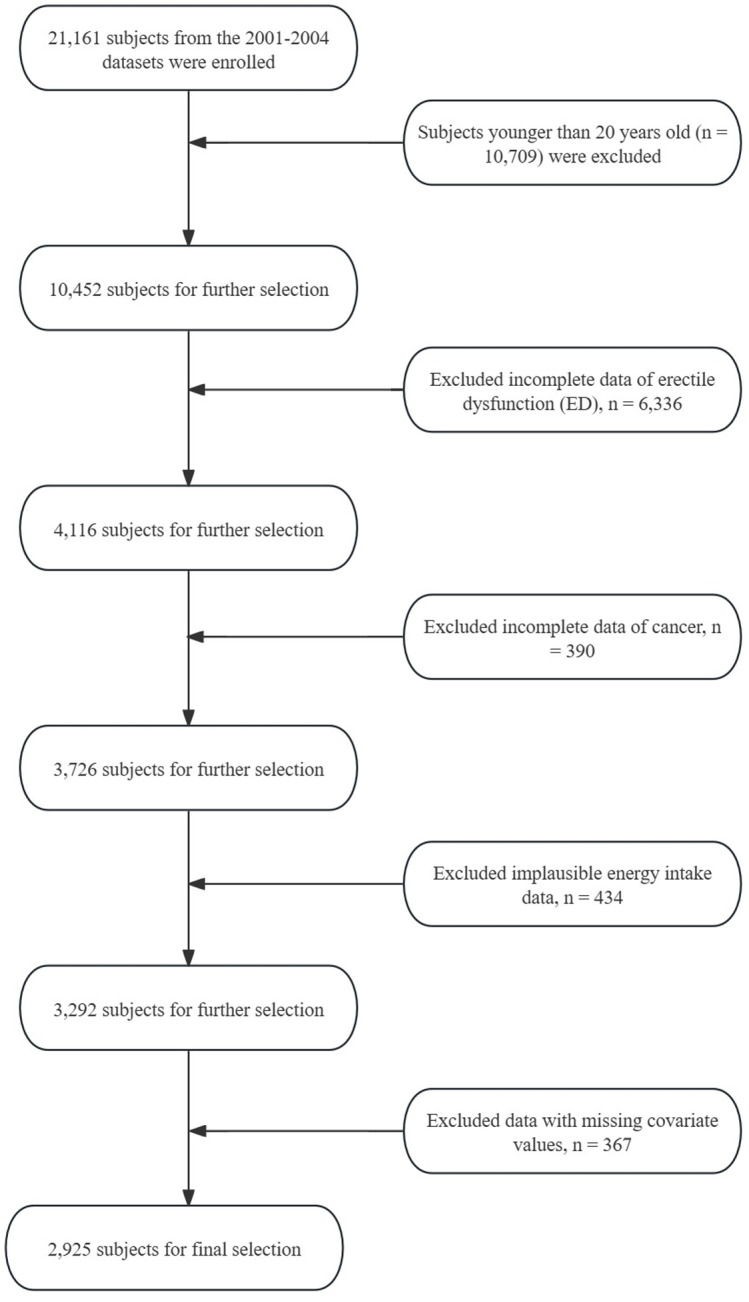
A flowchart showing the selection of study participants.

In our cross-sectional analysis of NHANES data from 2001 to 2004, we initially included 21,161 participants. Participants below the age of 20 years (10,709 individuals) were excluded. Additional exclusions were made for pregnant subjects (6,336), those with incomplete data on key indicators for ED, individuals with a history of cancer (390), and those with implausible energy intake data (434). The final step removed subjects with missing data on relevant covariates (367), resulting in a study population of 2,925 participants (Fig 1).

### 2.2. Ethical considerations

The National Health and Nutrition Examination Survey (NHANES) was conducted in accordance with the ethical standards established by the National Center for Health Statistics’ Ethical Review Committee. Prior to participation, all participants were provided with comprehensive information regarding the study’s aims, procedures, potential risks, and benefits. Written informed consent was obtained from each participant, ensuring their voluntary participation and the confidentiality of their personal and health information. The NHANES protocol was reviewed and approved by the NCHS Research Ethics Review Board, ensuring adherence to ethical guidelines and the protection of participants’ rights.

### 2.3. Data collection

#### 2.3.1. Exposure variable: Serum albumin (ALB).

Serum albumin levels were measured using the bromocresol purple (BCP) dye method. Serum specimens were processed, stored under refrigerated conditions (2–8 °C), and shipped to Collaborative Laboratory Services for testing and analysis. The DcX800 analyzer was employed, using a bichromatic digital endpoint method. In this method, albumin binds with the BCP reagent to form a complex, and the change in absorbance at 600 nm is monitored. This change in absorbance is directly proportional to the albumin concentration in the sample [23].

#### 2.3.2. Outcome variable: Erectile dysfunction (ED).

The assessment of erectile dysfunction (ED) was conducted using a private room at the Mobile Examination Center (MEC) with an audio computer-assisted self-interview (ACASI) format. ED was evaluated through a single question adapted from the Massachusetts Male Aging Study. Participants were asked to describe their ability to obtain and maintain an erection sufficient for satisfactory intercourse. The response options included: “always or almost always able,” “usually able,” “sometimes able,” and “never able.” For the purposes of this analysis, ED was defined as participants who responded “sometimes able” or “never able” to maintain an erection. Participants who answered “always or almost always able” or “usually able” were classified as not having ED [24,25].

#### 2.3.3. Covariate definitions.

Covariates were selected based on their potential impact on erectile function and included sociodemographic factors, lifestyle characteristics, and comorbid conditions.

**Sociodemographic Characteristics:** Participants’ demographic information was collected through self-administered questionnaires, which included details on age and race. Race was classified into four categories: Mexican American, Non-Hispanic White, Non-Hispanic Black, and Other. The poverty-to-income ratio (PIR), a measure of socioeconomic status, was calculated by dividing household income by the poverty threshold for the survey year and state, providing insight into the participants’ economic conditions. Marital status was categorized into two groups: cohabiting with a partner and living alone. Educational attainment was classified into three levels: less than high school, high school, and more than high school. Body Mass Index (BMI) was calculated using the formula: weight (kg)/ height squared (m²), and categorized into three groups: underweight/normal (< 25 kg/m²), overweight (25–30 kg/m²), and obese (≥ 30 kg/m²) [26].

**Lifestyle Characteristics:** Physical activity (PA) was measured in MET/h/week and categorized into three levels: less than 600, 600–8000, and more than 8000.Smoking status and Alcohol intaking status were categorized into three groups: never, former, and current. Dietary quality was assessed using the Healthy Eating Index (HEI) 2015. The HEI score ranges from 0 to 100, with higher scores indicating better dietary quality [27].

**Comorbid Conditions:** Diabetes was defined by any of the following: self-reported diagnosis, hypoglycemic medication use, fasting glucose ≥  7.0 mmol/L, random or 2-h OGTT glucose ≥  11.1 mmol/L, or HbA1c ≥ 6.5% [28]. Hypertension was defined as having a systolic blood pressure (BP) ≥ 140 mmHg, diastolic BP ≥ 90 mmHg, or current use of antihypertensive medication [29]. Hyperlipidemia was identified through specific lipid profile levels or the use of lipid-lowering medications [30].Cardiovascular disease (CVD) was defined as participants who had been previously diagnosed with congestive heart failure, coronary heart disease, angina, heart attack, or stroke [31].

### 2.4. Statistical analysis

The study population was stratified into quartiles based on serum albumin (ALB) levels. Continuous variables are reported as means ± standard errors (SE), whereas categorical variables are expressed as frequencies (%). To describe the characteristics of the study population, analysis of variance (ANOVA) was employed to compare continuous variables across ALB quartiles. For categorical variables, the Chi-square test. ALB levels were examined both as a continuous variable (per 1 g/dl change) and in a categorized format (quartiles).

The association between ALB levels and erectile dysfunction (ED) prevalence was investigated using weighted logistic regression models with the following adjustment strategies: Crude model (unadjusted);Model 1: Adjusted for age, race, poverty-to-income ratio (PIR), marital status, education level, and body mass index (BMI); Model 2: Adjusted for factors in Model 1 plus physical activity, smoking status, drinking status, and Healthy Eating Index (HEI-2015); Model 3: Adjusted for factors in Model 2 plus hypertension, hyperlipidemia, diabetes, and cardiovascular diseases. To assess the robustness of our findings against potential unmeasured confounding, we calculated E-values for both continuous and categorical analyses. To explore potential non-linear associations between ALB and ED, generalized additive models (GAMs) with smooth curve fitting were applied. Subgroup analyses and interaction effects were evaluated using weighted logistic regression across various demographic and clinical strata. All the analyses were performed with the statistical software packages R version 4.2.0 (http://www.R-project.org)), FreeStatistics software version 1.8, and EmpowerStats (http://www.empowerstats.com). P value < 0.05 was considered statistically significant.

## 3. Results

Of 2925 study participants, 747 were diagnosed with ED. Table 1 shows participant characteristics by serum albumin (ALB) quartiles. Participants in the highest ALB quartile were younger than those in the lowest quartile. The highest ALB quartile had more Non-Hispanic Whites, higher education levels, and lower obesity rates compared to the lowest quartile (all P < 0.001). They also had lower prevalence of chronic conditions including diabetes, hypertension, hyperlipidemia, cardiovascular diseases, and erectile dysfunction (all P < 0.001).

**Table 1:** Baseline characteristics of participants stratified by serum albumin quartiles.

variable	Q1 (2.9,4.2)	Q2 (4.2,4.4)	Q3 (4.4,4.5)	Q4 (4.5,5.5)	P-value
**Age** (years), mean (SE)	50.39 (0.63)	44.78 (0.68)	41.63 (0.85)	38.53 (0.57)	< 0.0001
**Race**, n (%)					< 0.0001
Mexican American	216 (6.69)	184 (8.29)	92 (9.70)	174 (9.82)	
Non-Hispanic Black	257 (13.94)	130 (8.26)	62 (9.08)	79 (5.53)	
Non-Hispanic White	509 (71.66)	429 (74.77)	193 (69.11)	399 (75.44)	
Other Race	59 (7.70)	50 (8.67)	32 (12.11)	60 (9.20)	
**PIR**, n (%)					0.09
Low (<1.5)	308 (19.87)	221 (19.30)	96 (17.13)	211 (19.31)	
Medium (1.5–3.5)	388 (36.85)	270 (32.73)	129 (29.08)	234 (31.44)	
High (>3.5)	345 (43.28)	302 (47.97)	154 (53.79)	267 (49.25)	
**Marital status**, n (%)					0.02
Cohabitation	748 (74.20)	582 (74.23)	260 (69.60)	452 (66.79)	
Solitude	292 (25.80)	211 (25.77)	119 (30.40)	259 (33.21)	
**Education level**, n (%)					< 0.001
Less than high school	325 (19.90)	220 (16.06)	90 (13.73)	165 (12.66)	
High school	251 (27.98)	200 (27.65)	103 (29.66)	157 (23.51)	
More than high school	464 (52.12)	372 (56.29)	186 (56.61)	390 (63.83)	
**BMI**, n (%)					< 0.0001
Normal (<25)	230 (20.30)	197 (24.84)	109 (28.42)	304 (41.38)	
Overweight (25 to < 30)	407 (39.00)	365 (44.62)	175 (44.07)	296 (41.22)	
Obese (30 or greater)	404 (40.70)	231 (30.54)	95 (27.51)	112 (17.40)	
**Physical activity** (MET/h/week), n (%)					0.3
>8000	9 (0.87)	3 (0.61)	1 (0.57)	3 (0.43)	
600–8000	276 (27.07)	222 (29.01)	102 (32.25)	220 (33.58)	
<600	756 (72.06)	568 (70.38)	276 (67.19)	489 (65.99)	
**Smoking status**, n (%)					< 0.0001
former	372 (33.28)	248 (27.60)	99 (25.74)	190 (24.50)	
never	356 (32.39)	340 (45.57)	179 (48.25)	332 (50.59)	
now	313 (34.33)	205 (26.83)	101 (26.01)	190 (24.90)	
**Alcohol intaking**, n (%)					< 0.0001
former	256 (22.02)	144 (13.98)	65 (13.67)	89 (10.96)	
never	79 (7.65)	54 (6.51)	23 (5.58)	43 (6.84)	
now	706 (70.33)	595 (79.51)	291 (80.75)	580 (82.20)	
**HEI-2015**,mean (SE)	47.88 (0.45)	48.92 (0.56)	49.62 (0.67)	50.20 (0.55)	0.01
**DM**, n (%)					< 0.0001
No	830 (83.66)	689 (90.13)	345 (93.72)	656 (95.38)	
Yes	211 (16.34)	104 (9.87)	34 (6.28)	56 (4.62)	
**Hypertension**, n (%)					< 0.0001
No	528 (58.05)	493 (66.91)	262 (72.61)	516 (73.27)	
Yes	513 (41.95)	300 (33.09)	117 (27.39)	196 (26.73)	
**Hyperlipidemia**, n (%)					< 0.001
No	256 (23.11)	183 (23.99)	94 (24.23)	244 (34.49)	
Yes	785 (76.89)	610 (76.01)	285 (75.77)	468 (65.51)	
**CVD**, n (%)					< 0.0001
No	850 (85.67)	703 (93.13)	345 (93.06)	671 (95.85)	
Yes	191 (14.33)	90 (6.87)	34 (6.94)	41 (4.15)	
**ED**, n (%)					< 0.0001
No	660 (72.04)	595 (83.36)	302 (86.97)	621 (92.09)	
Yes	381 (27.96)	198 (16.64)	77 (13.03)	91 (7.91)	

Abbreviations: PIR: Poverty Income Ratio; BMI: Body Mass Index; HEI-2015: healthy eating index-2015; DM: Diabetes Mellitus; CVD: cardiovascular diseases; ED: erectile dysfunction.

Continuous Variables: Presented as means with standard errors (SE); Categorical Variables: Displayed as counts (n) and percentages (%)

Table 2 shows associations between serum albumin levels and erectile dysfunction (ED). In the crude model, each 1 g/dL increase in albumin was associated with lower ED risk (OR: 0.13, 95% CI: 0.08–0.20, P < 0.0001). Participants in higher albumin quartiles (Q2–Q4) showed significantly lower ED risk compared to the lowest quartile (Q1). The association persisted after sequential adjustments. In Model 1 (adjusted for demographic and anthropometric factors), each 1 g/dL albumin increase was associated with lower ED risk (OR: 0.51, 95% CI: 0.28–0.93, P = 0.03), as was the highest quartile versus Q1 (OR: 0.55, 95% CI: 0.37–0.81, P = 0.01).Model 2 added lifestyle factors and maintained similar associations for both continuous albumin (OR: 0.55, 95% CI: 0.31–0.97, P = 0.04) and Q4 versus Q1 (OR: 0.56, 95% CI: 0.38–0.83, P = 0.01). The final model, adjusting for comorbidities, showed consistent results for continuous albumin (OR: 0.53, 95% CI: 0.29–0.97, P = 0.04) and Q4 versus Q1 (OR: 0.56, 95% CI: 0.35–0.90, P = 0.02). For sensitivity analysis of unmeasured confounding, the E-values were 2.09 (continuous analysis) and 2.01 (highest vs. lowest quartile comparison), suggesting substantial unmeasured confounding would be needed to nullify the observed associations.

**Table 2:** Association between serum albumin levels and erectile dysfunction across different regression models.

	crude model	Model 1	Model 2	Model 3
character	OR (95%CI) P-value	OR (95%CI) P-value	OR (95%CI) P-value	OR (95%CI) P-value
ALB (per 1 g/dl change)	0.13 (0.08,0.20) <0.0001	0.51 (0.28,0.93) 0.03	0.55 (0.31,0.97) 0.04	0.53 (0.29,0.97) 0.04
ALB quartile				
Q1	ref	ref	ref	ref
Q2	0.51 (0.39,0.68) < 0.0001	0.78 (0.52,1.15) 0.19	0.79 (0.53,1.18) 0.23	0.83 (0.52,1.31) 0.37
Q3	0.39 (0.27,0.56) <0.0001	0.79 (0.53,1.19) 0.24	0.82 (0.54,1.22) 0.30	0.89 (0.58,1.36) 0.54
Q4	0.22 (0.16,0.30) < 0.0001	0.55 (0.37,0.81) 0.01	0.56 (0.38,0.83) 0.01	0.56 (0.35,0.90) 0.02
p for trend	<0.0001	0.01	0.01	0.03

OR, odds ratio; 95% CI, 95% confidence interval.

Crude: No covariates adjusted.

Model 1: Adjusted for age, race, poverty-to-income ratio (PIR), marital status, education level, and body mass index (BMI).

Model 2: Includes all adjustments from Model 1, with additional adjustments for physical activity, smoking status, drinking status, and Healthy Eating Index (HEI-2015).

Model 3: Extends the adjustments of Model 2 to include hypertension, hyperlipidemia, diabetes, and cardiovascular diseases.

**Fig 2:**
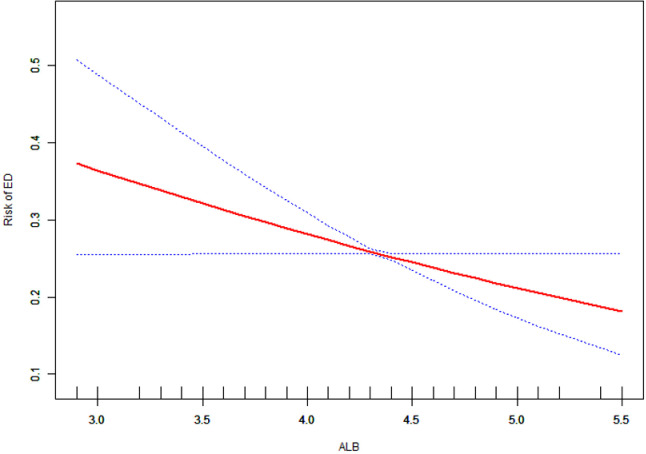
Smoothed curve fitting of serum albumin levels and erectile dysfunction risk. Figure Annotation: This generalized additive model (GAM) plot illustrates the relationship between ALB concentration (X-axis) and the Risk of ED (Y-axis). The red curve represents the fitted line, while the blue shaded area denotes the 95% confidence interval.

Fig 2 presents a curve fitting analysis using a generalized additive model (GAM) to elucidate the relationship between serum albumin (ALB) levels and erectile dysfunction (ED). This analysis adjusts for a wide array of confounders, including demographic factors (age, race, poverty-to-income ratio (PIR), marital status, education level, and body mass index (BMI)), lifestyle factors (physical activity, smoking status, drinking status, and Healthy Eating Index (HEI-2015)), and prevalent comorbid conditions (hypertension, hyperlipidemia, diabetes, and cardiovascular diseases). The GAM-derived curve clearly demonstrates an inverse correlation between ALB levels and ED risk, with no significant inflection points, indicating a consistent linear relationship.

Table 3 presents the stratified analysis and interaction tests of serum albumin (ALB) levels and erectile dysfunction (ED) risk among U.S. adults from NHANES 2001–2004. The results indicate no significant interaction between ALB levels and age, race, BMI, diabetes status, hypertension, hyperlipidemia, or cardiovascular disease (CVD). Overall, the inverse association between higher ALB and lower ED risk was consistently observed across strata, although the strength of this association appeared to vary by certain demographic and clinical factors.

**Table 3:** stratified analysis and interaction tests of serum albumin levels and erectile dysfunction risk in U.S. adults: NHANES 2001–2004.

character	OR (95%CI) P-value	p for interaction
**Age**		0.821
20–45 years	0.316 (0.078, 1.276), 0.096	
45–65 years	0.636 (0.247,1.633), 0.310	
65–85 years	0.600 (0.269,1.341), 0.188	
**Race**		0.269
Non-Hispanic White	0.719 (0.335,1.545), 0.366	
Other Race	0.069 (0.002, 2.051), 0.102	
Non-Hispanic Black	0.445 (0.154,1.287), 0.117	
Mexican American	1.093 (0.543, 2.201), 0.791	
**BMI**		0.523
Normal (<25)	0.466 (0.127,1.715), 0.221	
Overweight (25 to < 30)	0.564 (0.234, 1.357), 0.177	
Obese (30 or greater)	0.746 (0.241,2.311), 0.576	
**DM**		0.963
No	0.674 (0.333,1.365), 0.244	
Yes	0.475 (0.172,1.309), 0.134	
**Hypertension**		0.767
No	0.606 (0.220,1.665), 0.298	
Yes	0.529 (0.281,0.998), 0.049	
**Hyperlipidemia**		0.195
No	0.658 (0.203,2.133), 0.450	
Yes	0.544 (0.307,0.963), 0.039	
**CVD**		0.471
No	0.532 (0.267,1.062), 0.070	
Yes	0.832 (0.207, 3.351), 0.777	

OR, odds ratio; 95% CI, 95% confidence interval. ALB was analyzed as a continuous variable. The P for interaction was calculated using weighted multivariable logistic regression analysis. This analysis was adjusted for age, race, poverty-to-income ratio (PIR), marital status, education level, body mass index (BMI), physical activity, smoking status, drinking status, Healthy Eating Index (HEI-2015), hypertension, hyperlipidemia, diabetes, and cardiovascular diseases, excluding the specific subgroup variable.

## 4. Discussion

In this nationally representative NHANES 2001–2004 study, we demonstrated an inverse relationship between serum albumin levels and erectile dysfunction (ED) risk after controlling for confounders. Smooth curve fitting revealed a linear dose-response relationship.

Our findings corroborate and extend previous clinical evidence indicating an inverse correlation between serum albumin levels and the risk of erectile dysfunction (ED). Compared to Yamamoto’s study which focused on Japanese ulcerative colitis patients with severe ED, our NHANES-based analysis examined the general U.S. population across all ED severity levels. Our methodology was more comprehensive, using multiple adjustment models and quartile analysis versus their binary albumin classification (low vs. normal). While both studies found significant associations between low albumin and ED, our larger sample size and detailed analytical approach provided stronger evidence for a dose-response relationship [12]. Our findings align with Mohamed Fakhry’s study on cirrhotic patients, which also found low albumin levels to be a significant predictor of ED. However, their study focused on a specific population with liver disease and used more comprehensive ED assessment methods, including both IIEF-5 questionnaire and penile Doppler. While they reported a higher ED prevalence (80%) likely due to their cirrhotic population, both studies consistently demonstrate the importance of albumin in erectile function [32].

Higher plasma albumin levels may reduce the risk of erectile dysfunction in men through multiple pathways. First, moderately elevated albumin can bind and stabilize nitric oxide (NO), a key vasodilator molecule that maintains penile erection, thereby enhancing NO-mediated relaxation of smooth muscles in the penile corpora cavernosa and facilitating cavernosal engorgement, which is beneficial for erection [33,34]. Second, albumin possesses antioxidant and anti-inflammatory properties, which can alleviate the damage caused by reactive oxygen species and free radicals to endothelial cells, preserve endothelial cell function, and maintain the vasodilatory capacity of the penile corpora cavernosa [35–37]. Moreover, albumin may participate in the transport and regulation of androgens, indirectly maintaining appropriate levels of free testosterone and other androgens, which is conducive to maintaining normal sensitivity of the erectile reflex [38]. Theoretically, moderately elevated plasma albumin levels are likely to improve the hemodynamic regulation required for penile erection through the aforementioned pathways, thereby reducing the risk of erectile dysfunction. However, the specific molecular mechanisms by which albumin affects erectile function require further in-depth research to elucidate.

While our findings suggest a relationship between albumin levels and ED risk, alternative explanations should be considered. To address potential confounding, we adjusted for major comorbidities including hypertension, hyperlipidemia, diabetes, and cardiovascular diseases. The E-value of 2.09 for our primary finding suggests that substantial unmeasured confounding would be needed to nullify the observed association. Nevertheless, given the cross-sectional design, we cannot completely rule out the possibility that unmeasured factors might simultaneously influence both parameters.

Our study has several notable strengths. First, we utilized data from NHANES, a large multi-ethnic, nationally representative database, which enhances the generalizability of our findings. Second, our comprehensive statistical approach included extensive covariate adjustment across multiple models and sensitivity analyses, strengthening the robustness of our results. Third, we innovatively employed generalized additive model (GAM) plotting to visualize the dose-response relationship between ALB and ED risk. Fourth, our thorough subgroup analyses across various demographic and clinical characteristics confirmed the consistency of findings across different populations. Finally, compared to previous hospital-based studies, our population-based design minimizes selection bias.

However, our study has certain limitations that warrant acknowledgment. First, given the cross-sectional nature of this study, we cannot establish causality between albumin levels and erectile dysfunction (ED). Second, the assessment of ED relied on self-reported data from survey participants, which may have led to an underestimation of the true ED prevalence. While we adjusted for numerous potential confounders, residual confounding from unmeasured or inaccurately measured variables cannot be ruled out, necessitating cautious interpretation of the observed albumin-ED risk relationship. Moreover, our analysis of NHANES 2001–2004 data, though selected due to the specific ED assessment module availability during this period, may not fully reflect current population characteristics and disease patterns. Lastly, as the NHANES database represents the U.S. population, the generalizability of our results to other national or regional populations needs to be further verified through additional studies.

Our findings have direct clinical relevance. The demonstrated inverse relationship between serum albumin and erectile dysfunction suggests that albumin assessment, a routine laboratory measurement, should be considered in ED evaluation. While further research is needed to establish causality, monitoring and maintaining optimal albumin levels may represent a potential therapeutic approach in ED management. This readily available laboratory parameter could enhance current clinical approaches to ED prevention and treatment.

## 5. Conclusion

In summary, our examination of NHANES data demonstrates a significant inverse relationship between ALB and ED risk. This relationship underscores the importance of ALB in the context of ED and suggests the potential for modifications to influence ED risk. The cross-sectional nature of our study calls for further longitudinal investigations to explore these associations in depth and to evaluate the effectiveness of specific interventions.
